# An automatic UPLC‐HRMS data analysis platform for plant metabolomics

**DOI:** 10.1111/pbi.13180

**Published:** 2019-06-17

**Authors:** Pingping Liu, Huina Zhou, Qingxia Zheng, Peng Lu, Yong‐Jie Yu, Peijian Cao, Wei Chen, Qiansi Chen

**Affiliations:** ^1^ Zhengzhou Tobacco Research Institute of CNTC Zhengzhou Henan China; ^2^ College of Pharmacy Ningxia Medical University Yinchuan Ningxia China; ^3^ National Key Laboratory of Crop Genetic Improvement Huazhong Agricultural University Wuhan Hubei China

**Keywords:** ion-clustering peak annotation, plant metabolomics, UPLC‐HRMS, chemometrics

Here, we want to introduce our new automatic data analysis platform for untargeted metabolomic analysis of complex plant samples. Many laboratories across the world have adopted ultra‐high performance liquid chromatography‐high resolution mass spectrometry (UPLC‐HRMS) as they seek to thoroughly characterize metabolites in complex plant samples, with the larger aim of identifying compounds with impactful biological functions (Rinschen *et al*., 2019; Shen *et al*., 2019). Despite the widespread deployment of analytical hardware that is capable of capturing robust data sets for many types of biological samples, it is the data analysis steps for UPLC‐HRMS – seeking to accurately extract qualitative and quantitative information for thousands of metabolites – that continues to be one of the most challenging tasks and has become a frustrating bottleneck for metabolomics and lipidomics (Domingo‐Almenara *et al*., 2018).

A number of proprietary and freely available methodologies have been developed for such analyses, including AntDAS (Fu *et al*., 2017), Mzmine2 (Pluskal *et al*., 2010), XCMS (Smith *et al*., 2006) and MS‐DIAL (Tsugawa *et al*., 2015). These methods typically integrate the entire data analysis workflow for untargeted metabolomics (i.e. EIC construction, peak detection, peak annotation and peak alignment) and connect to chemometrics methods for screening out functionally impactful metabolites that exhibit significant differences amongst various experimental groups.

In practical applications, especially for complex plant sample analysis, however, researchers are still faced with the challenge that many identified signals can correspond to the same biological metabolite, because hundreds of ions can screen out based on analysis of variance (ANOVA) or popular algorithms in chemometrics like partial least square. Aiming to address this problem, researchers seeking to perform compound identification must manually identify ions that putatively originate from a single metabolite (e.g. neutral loss and fragment ions), which is certainly a very time‐consuming task. Aiming to address this problem, we have developed an ion clustering‐based fragment identification algorithm. We combine this new algorithm with our previously developed data analysis methods (Fu *et al*., 2016a; Yu *et al*., 2019), including peak detection and time shift correction and registration modules, to provide an integrated data analysis platform for UPLC‐HRMS‐based untargeted metabolomics. The platform comprises five modules, including one for EIC peak extraction, one for time shift correction, one for peak registration across samples, one for peak screening module and finally our ion clustering‐based peak annotation module (a full flow chart of the platform is presented in Figure 1).

**Figure 1:**
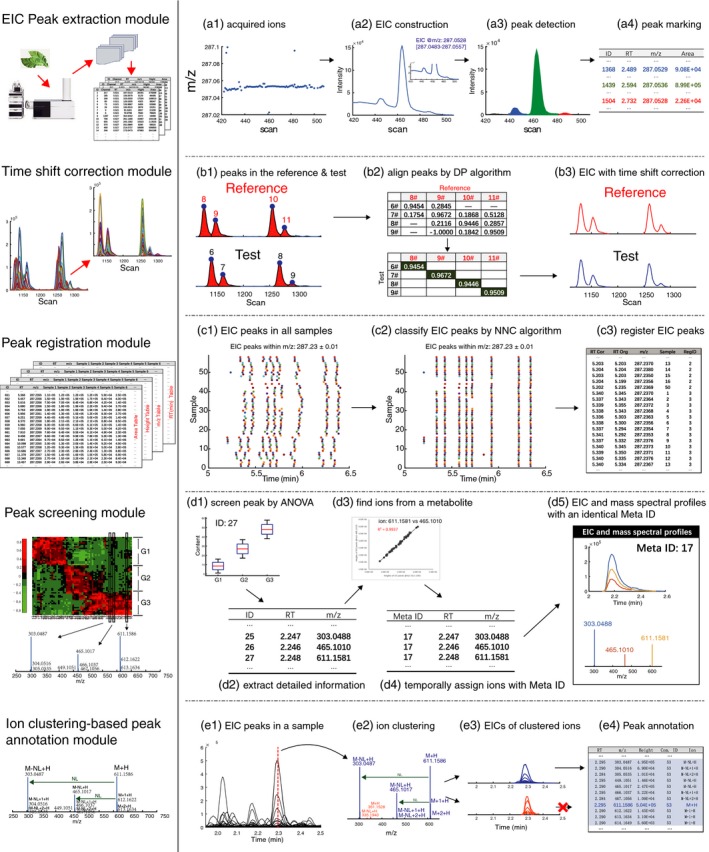
Workflow of our platform for automated untargeted metabolomics data analysis for plant samples. In the peak extraction module, the ion density of a metabolite will be higher than background noise (a1) and an EIC is extracted with a density clustering algorithm (a2), which is then used for peak detection after baseline correction (c1). All detected peaks in a sample are marked based on *m/z* values and are put into an EIC peak list table. In the time shift correction module, peaks in test and reference EICs are marked (b1) to build a similarity matrix (b2). A dynamic programming algorithm is then used to search aligned nodes in the matrix to perform time shift correction (b3). In the peak registration module, EIC peaks in all samples are marked (c1) and registered based on a nearest neighbouring connecting algorithm to group those from the same ion (c2). Peaks in a group are registered with the same identifier (c3). In the Peak screening module, EIC peaks are screened based on an inferential statistical analysis like ANOVA (d1), and their detailed information like retention times and *m/z* values is extracted (d2). Linear relationships for each pair of peaks are determined based on their peak heights across samples (d3). The EIC peak pair will be temporally assigned the same ‘Meta ID’ if they obtained a high correlation coefficient (d4). (d5) Mass spectrum and EIC profiles for ions with an identical ‘Meta ID’. In the ion clustering‐based peak annotation module, all EIC peaks in a sample are provided in the retention time axis (e1), and these are clustered based on their peak shape similarities and retention time differences (e2). (e3) shows EIC profiles of ions in a cluster. Fragment ions, together with isotopic ions and adduct ions of screened metabolites, are finally annotated (e4).

## EIC peak extraction module

The acquired UPLC‐HRMS data files from an instrument are transformed into *mzxml* file format using ProteoWizard (http://proteowizard.sourceforge.net/). EICs are constructed using an ion density clustering algorithm(Yu *et al*., 2019), which extracts EICs based on the fact that ions from a metabolite exhibit almost identical *m/z* values so that, within a small *m/z* tolerance like 0.01 Da, the specific ion density will be higher than any particular background noise signal. Peak detection is performed for each EIC. First, a local‐minimal value‐based baseline drift correction algorithm (Fu *et al*., 2016b) is introduced for baseline correction.

Next, chromatographic peaks are extracted using a Gaussian smoothing‐based strategy (Fu *et al*., 2016a), which extracts peaks by smoothing the EIC under different smoothing scales; it then searches the ridge lines across successively increased smoothing scales. An example of peak extraction is provided in Figure 1a1‐a3, where three chromatographic peaks in the EIC have been extracted. Then, retention time and *m/z* value of the ion acquired at the peak apex are used to characterize each EIC peak (Figure 1a4). Quantitative information based on peak area and peak height is extracted, respectively, as the sum of responses in the elution EIC peak and as the intensity at the peak apex.

## Time shift correction module

Time shift correction is performed for each EIC. First, the total number of peaks in each EIC is counted, and the EIC with the maximum number of components is selected as the reference sample (Figure 1b1). For each peak in the reference, its candidate traces in a test sample must satisfy both *m/z* and retention tolerances, which are set as 0.01 Da and 0.5 min, respectively. The similarity between a test peak and a reference peak is assessed based on the Pearson correlation coefficient values of EIC curves. A similarity matrix can thus be constructed based on the test and reference peaks (Figure 1b2). We align all reference peak simultaneously by searching an optimization path based on maximizing accumulated Pearson coefficients in the matrix. An advantage is that it is insensitive to peaks with similar elution profiles. The modified dynamic programming (DP; Fu *et al*., 2017) is introduced for this purpose. Nodes in the optimization path from the DP represent aligned peaks. The time shift values for a given EIC are estimated based on these aligned peaks by using the linear interpolation function in MATLAB. The aligned EIC in Figure 1b3 suggests that the time shift problem in the test sample has been accurately resolved.

## Peak registration module

Our peak registration module classifies EIC peaks from each of the different samples into a single ion group if they correspond to the same ion. We here developed a nearest neighbouring connecting (NNC) algorithm (Yu *et al*., 2019) to register EIC peaks. The NNC uses the modified retention time of each EIC peak that is obtained from the *Time shift correction module*. For each peak, its candidate peaks are determined as those satisfying both *m/z* tolerance (0.01 Da) and retention time tolerance (0.1 min) limits, and the candidate peaks are presented according to their retention time differences in an ascending order.

Each connected pair ([*EIC peak*,* first candidate*]) is scanned by the NNC algorithm. If the role in the pair can be changed, that is *EIC peak* is the first candidate peak of the *first candidate*, peaks in the pair will be classified into a group. A constraint condition of our NNC algorithm is that a group cannot include more than one EIC peak from the same sample. The scanning procedure of NNC iteratively repeats until EIC peaks cannot be classified further. At last, EIC peaks in an NNC group are registered with the same identifier in the registered component table (Figures 1c1‐c3).

## Peak screening module

Our strategy first screens metabolites using ANOVA to identify peaks with statistically significant differences in mean values amongst sample groups (Figures 1d1‐d2). Due to the complexity of sample composition frequently encountered with plant samples, the situation that several screened ions come from the same metabolite is frequently encountered. This problem increases the complexity of metabolite identification and reduces the data analysis efficiency. Theoretically, a bilinear structure can be obtained for the ions from a single metabolite. Thus, a strong linear correlation for peak height or peak area values can be identified for ions that are truly from a single metabolite. (Figure 1d3).

The Pearson coefficient between two screened peaks is calculated based on peak heights. A minimum covariance determinate is used to eliminate the influence of outlying samples. If the coefficient between two screened ions is above a user‐defined value, such as 0.9, the ions will be temporally identified as being from a single metabolite and are marked with an identical ‘Meta ID’ (Figure 1d4). Examples of EIC and mass spectral profiles of ions with identical ‘Meta ID’ are shown in Figure 1d5.

## Ion clustering‐based peak annotation module

Our platform will further verify whether or not the ions with identical ‘Meta ID’ come from the same metabolite. We developed a bottom‐up ion‐clustering algorithm that iteratively identifies which ions are likely to originate from the same metabolite. It first identifies isotopic ions, that is [M+H]^+^ and [M + 1 + H]^+^ from a putative compound based on an initialized EIC peak shape similarity cut‐off value (0.9) and retention time tolerance (0.02 min). The EIC peak shape similarity cut‐off value for each [M+H]^+^ ion will be adaptively determined based on its isotopic ions. Then, other types of ions like isotopic ions (such as [M + 2 + H]^+^, [M + 3 + H]^+^) and/or adduct ions (K^+^, Na^+^, NH_4_^+^, etc.) are recognized.

The [M+H]^+^ ions are then clustered based on their peak shapes and retention times. If [M+H]^+^ ions in a sample are identified as originating from a single metabolite, the platform then returns to the *Peak screening module* to verify if they have been screened and grouped with the identical ‘Meta ID’. Given that the ions have been clustered both within a sample based on peak shapes and across samples based on peak heights, they will be ultimately identified as being from a single metabolite. Finally, the vector values for the peaks comprising a metabolite are concatenated to generate a derived mass spectrum for the metabolite.

Figure 1e1 shows EIC peaks in a sample and ion distribution characteristics of EIC peaks, with peak apexes at about 2.29 min shown in Figure 1e2. Ions in the range from 300 to 620 Da are classified into two clusters based on our ion‐clustering algorithm; these are separately marked as red and blue lines for visualization (Figure 1e2). One can observe from Figure 1e3 that tightly coeluted ions which come from different metabolites can be satisfactorily deconvoluted with our platform. Based on combination with the *Peak screening module* results, we can see that the ions in the bottom plot of Figure 1e3 are not significantly different amongst sample groups. Finally, the ions in a given cluster are annotated as being from a screened single metabolite, that is rutin in Figure 1e; each ion is labelled with the identifier for a putative single metabolite Figure 1e4.

These five modules work together to automate UPLC‐HRMS data analysis. A significant advantage of this platform is that the screened fragment ions from the same metabolite can be accurately identified automatically using information from both chromatographic profiles and peak height relationships amongst samples. Notably, there is no requirement for any data transformation amongst the different modules for identifying fragment ions, a major difference from many of the publicly available untargeted metabolomics data analysis tools. Moreover, fragment ion identification is organically integrated into our platform. We implemented our platform in a MATLAB GUI, which is freely available at: http://software.tobaccodb.org/software/PlantMetAnal.

## Conflicts of interest

The authors declare no conflict of interest.
